# A Case of Polyarticular *Pasteurella multocida* Septic Arthritis

**DOI:** 10.1155/2016/5025697

**Published:** 2016-04-11

**Authors:** Sarah Nitoslawski, Todd M. McConnell, Makeda Semret, Michael A. Stein

**Affiliations:** ^1^McGill University, Montreal, QC, Canada H3A 2T5; ^2^Division of General Internal Medicine, St. Mary's and Royal Victoria Hospital, McGill University, Montreal, QC, Canada H3T 1M5; ^3^Division of Infectious Diseases, St. Mary's Hospital, McGill University, Montreal, QC, Canada H3T 1M5; ^4^Division of Rheumatology, St. Mary's and Royal Victoria Hospital, McGill University, Montreal, QC, Canada H3T 1M5

## Abstract

A 76-year-old man with a history of osteoarthritis presents with right leg erythema and inability to weight-bear and pain in his right shoulder. Synovial fluid cell count of the knee and shoulder showed abundant neutrophils, and cultures of the knee showed growth of *Pasteurella multocida*. The patient owned four cats with which he had frequent contact, but history and physical examination elicited no evidence of scratches or bites. This case highlights the invasive potential of *Pasteurella multocida* in an immunocompetent individual and its capacity to cause septic arthritis in the setting of frequent animal contact.

## 1. Introduction

*Pasteurella multocida*, a small Gram-negative coccobacillus, is part of the commensal oral-pharyngeal flora in many domestic animals, notably dogs (isolated in 50–60% of cultures) and cats (isolated in 70–90% of cultures) [1, 2]. Human infections with* P. multocida* most commonly occur following animal bites or scratches but may occur in the absence of trauma, likely secondary to contact with the animal's secretions [3, 4]. Local wound infections from an animal bite are the most common human infections caused by* P. multocida* [4–6]. Rarely,* P. multocida* may also be more responsible for more serious human infections, such as septic arthritis, particularly in joints previously damaged by rheumatoid arthritis or osteoarthritis [7, 8].

## 2. Case Presentation

A 76-year-old male presented to the emergency room with a ten-day history of unilateral erythema and edema of the right leg and a two-day history of right knee pain and inability to weight-bear. He had experienced fever and rigors the day before, along with some “delirious” behavior, according to a family member. His past medical history was significant for hypertension, dyslipidemia, and bilateral osteoarthritis of the knees. There was a remote history of reactive arthritis in the 1970s with an unknown infectious cause.

He was afebrile on presentation. His white blood cell count was elevated at 16.2; CRP was markedly elevated at >380 mg/L; his ESR was normal at 19 mm/h. Blood cultures and synovial fluid from the knee were collected, and he was started on cefazolin 2 g every 8 hours. Upon his admission to the medical ward, the patient was delirious and also complaining of significant right shoulder and right ankle pain. Both joints were tender to touch and warm with restricted range of motion. Synovial fluid was drained from both the shoulder and the ankle on the second day of admission. An immunoglobulin panel was drawn to rule out possible immune deficiency, including multiple myeloma. The patient continued to be very confused; therefore, CT brain and lumbar puncture were performed. Total body bone and gallium, as well as imaging of the ankle and knee, were performed several days later.

## 3. Diagnosis

Synovial fluid from the knee and the shoulder showed abundant neutrophils (133 995 × 10^6^ per liter for the knee and 621 528 × 10^6^ per liter for the shoulder), consistent with multifocal septic arthritis. Only a small amount of synovial fluid could be collected from the ankle joint. The patient underwent debridement of the knee followed by debridement of the shoulder the following day.

Gram-negative rods were seen on microscopy of synovial fluid from the knee, but not from other specimens. After 48 hours, the knee synovial fluid culture showed growth of* Pasteurella multocida*, sensitive to ampicillin (MIC < 2), cefazolin (MIC < 2), ceftriaxone (MIC < 1), and ciprofloxacin (MIC < 0.25). Upon further questioning, the patient admitted to owning four cats, with which he had close physical contact, but denied any trauma in the form of bites or scratches. The blood cultures, although they were collected before the initiation of antibiotics, yielded no growth. Because there was a suspicion of central nervous system involvement, cefazolin was changed to Penicillin G 4 million units every 4 hours for optimal CNS penetration.

Culture of fluid from the inflamed shoulder and ankle showed no growth; however these had been collected approximately 48 hours after initiation of antibiotics. The immunoglobulin panel was normal.

Bone and gallium scan of the body and lower extremities showed heterogeneous uptake in the right shoulder, right knee, and right ankle compatible with active infection. CT of the right ankle showed a moderate ankle joint effusion with marginal erosions within the subtalar joint, suggesting involvement of the subtalar joint as well. MRI of the right knee showed severe tricompartmental osteoarthritic changes with extensive inflammatory changes and fat stranding in the soft tissues surrounding the knee.

CT brain was normal. CSF showed 780 × 10^6^ per liter red blood cells but no leukocytes and no bacterial growth, eliminating* Pasteurella* meningitis as a cause of delirium. Syphilis serology was negative. Penicillin G was changed to ceftriaxone 2 g every 12 hours. The patient's confusion persisted throughout his stay in hospital and ultimately resolved in a rehabilitation center. His arthritis, however, persisted following a six-week course of antibiotics, which was completed with ciprofloxacin. In fact, the patient developed significant ongoing bilateral synovitis of his wrists and metacarpophalangeal joints, as well as his ankle and knee joints. The patient remained seronegative for rheumatoid factor, consistent with a reactive arthritis, and was treated with tapering oral corticosteroids and methotrexate.

## 4. Discussion

Septic arthritis due to* Pasteurella multocida* is rare and most commonly occurs by direct percutaneous inoculation following penetrating animal bites [5, 9]. The case described is unusual, because there was no evidence of trauma on history, although the patient did admit to frequent exposure to at least four cats. Upon further probing, the patient did admit to often allowing the cats to lick his skin. We postulate that* P. multocida* infection and subsequent septic arthritis occurred due to frequent contact with the cats' saliva. Infection in humans may occur with no apparent animal contact in approximately 5–10% of cases [3, 10]. Contiguous spread to a joint from a local bite or scratch is the most common route of infection, and hematogenous dissemination is considered rare [4, 11–13]. Indeed, Weber et al. found 47 reported cases of* P. multocida* bacteremia, with few reports since then, other than in three immunocompromised patients [3, 14]. In this case the infection involved three distant sites (ankle, knee, and shoulder), which strongly suggests that the spread was through the hematogenous route, even though the patient was not immunosuppressed.

*P. multocida* septic arthritis seems to have a predilection for previously damaged joints, with cases reported in patients with rheumatoid arthritis or prosthetic joints [3, 15, 16]. There have been 22 cases reported of prosthetic joint infections with* P. multocida* [14]. In our case, the patient had a long-standing history of bilateral severe knee osteoarthritis and a remote history of reactive arthritis of his knee, but he was not known for degenerative joint disease in the shoulders or ankles. Polyarticular involvement in* P. multocida* septic arthritis is quite unusual; a single joint is affected 88% of the time [3, 17]. Most previously reported cases of polyarticular septic arthritis due to* P. multocida* occurred in the context of underlying systemic illness, such as rheumatoid arthritis on steroids, severe liver disease, and end-stage renal disease [7, 8, 12, 13]. However, our patient had no such predisposition for polyarticular* P. multocida* septic arthritis, as he had no prior systemic chronic illness nor was he immunocompromised.

This case report highlights the invasive potential of* P. multocida* even in patients without overt immunosuppression and particularly its capacity to cause polyarticular septic arthritis in the setting of frequent animal contact. It suggests a potential association between osteoarthritis and* P. multocida* joint infection, which should be further explored.
